# Pharyngeal-Esophageal Malignancies with Dermatologic Paraneoplastic Syndrome

**DOI:** 10.3390/life12111705

**Published:** 2022-10-26

**Authors:** Ana Fulga, Doriana Cristea Ene, Laura Bujoreanu Bezman, Oana Maria Dragostin, Iuliu Fulga, Elena Stamate, Alin Ionut Piraianu, Florin Bujoreanu, Alin Laurentiu Tatu

**Affiliations:** 1 Faculty of Medicine and Pharmacy, Dunarea de Jos University of Galati, 35 AI Cuza st, 800010 Galati, Romania; 2 Saint Apostle Andrew Emergency County Clinical Hospital, 177 Brailei st, 800578 Galati, Romania; 3 Cardiology Department, University Emergency Hospital Bucharest, 169 Independence Square st, 050098 Bucharest, Romania; 4 Saint Parascheva Clinical Hospital of Infectious Diseases, 393 Traian st, 800179 Galati, Romania

**Keywords:** paraneoplastic syndrome, skin manifestations, neoplasm, pharynx, esophagus

## Abstract

Systemic changes often send signals to the skin, and certain neoplastic diseases of the internal organs can also trigger skin manifestations. In this article, the authors make clinical photography presentations of the patients seen at our clinic with dermatologic paraneoplastic syndromes within pharyngeal–esophageal malignancies, describe several paraneoplastic dermatoses, and also review high-quality scientific literature in order to be able to highlight the dermatological signs of pharyngoesophageal malignant tumors. The majority of our patients with paraneoplastic dermatoses, filtering for pharyngoesophageal malignancies, had esophageal neoplasms, out of whom seven were female and two were male, making esophageal cancer more common within the paraneoplastic dermatoses within pharyngoesophageal malignancies. An early recognition of paraneoplastic dermatoses can diagnose neoplasms and sequentially contribute to a better prognosis for the patient. This matter is also useful for front-line medical personnel in order to improve early diagnosis of the underlying malignancy, curative interventions with prompt therapy administration and good prognosis.

## 1. Introduction

Paraneoplastic diseases are disorders of hematological, endocrine, or nervous system as well as clinical and biochemical imbalances that are related to the existence of malignant tumors but are not directly linked to the tumor invasion or the metastasis of the original tumor [1]. The skin may also provide a clinician with signs that are suggestive of systemic diseases, thus contributing to the diagnosis of many diseases, including malignant tumors [2,3].

Globally, esophageal cancer is one of the leading causes of cancer mortality, being responsible, together with other gastrointestinal cancers, for about 1/3 of all disability adjusted life-years (DALYs) from cancer [4,5]. In the last almost 20 years, the incidence of this pathology has increased for the new cases by 52.3%, from 310,000 to 473,000, the number of deaths increased by 40%, from 311,000 to 436,000, and the total DALYs increased by 27.4%, from 7,680,000 to 9,780,000 [6]. If we refer to esophageal adenoma, in the last 30 years, its incidence has increased faster than of any other solid tumor in the Western world, with an increase of 500% [7], and the five-year survival rates remain unacceptably low, at only 10%, reflecting late addressability for esophageal tumors [8].

The low survival rate of patients with esophageal carcinoma and the disabling nature of the specific paraneoplastic syndrome obliges the scientific community to a prompt systemic response to combat the phenomenon, which requires major efforts regarding early identification and reduction of risk factors, focusing on the problem through the appropriate allocation of material and human resources, as well as the development of effective methods of prediction and prophylaxis by monitoring the categories of patients with predisposing factors [9].

In this article, the authors present clinical photographs of patients with dermatologic paraneoplastic syndromes within pharyngeal–esophageal malignancies, describe several of these paraneoplastic dermatoses, and review high-quality scientific literature to be able to highlight the dermatological signs of pharyngoesophageal malignant tumors useful for front-line medical personnel in order to improve the early diagnosis of the underlying malignancy, curative interventions with prompt therapy administration and good prognosis.

## 2. Methods

A literature search was conducted for articles published in the English language in the ScienceDirect, SpringerLink and PubMed electronic database. The keywords used for our research purposes were “paraneoplastic syndrome”, “skin manifestations”, “neoplasm”, “pharynx”. Furthermore, we analyzed clinical photographs of nine patients with paraneoplastic dermatoses and pharyngeal–esophageal malignancies over a period of 10 years dating 2012–2022 from the Saint Parascheva Clinical Hospital of Infectious Diseases and the Saint Apostle Andrew Emergency County Clinical Hospital from Galati, Romania, with the patient consent forms containing patient details and/or images signed by all the patients.

## 3. Relevant Literature

The original articles written in English were found in Web of Science, Science Direct, and Springer Link using the following keywords: paraneoplastic, skin manifestations, neoplasm, pharynx, esophagus. In the database, 13 papers regarding skin manifestations in pharyngoesophageal neoplasms were identified [2,3,10,11,12,13,14,15,16,17,18,19,20,21,22], which are all shown in Table 1.

## 4. Discussion

The pharyngoesophageal junction, also called “the mouth of the esophagus” [45], represents the passage from the hypopharynx to the cervical esophagus (C5–C6 vertebral interspace, inferior cricoid cartilage border) and is a high intraluminal pressure area that serves as a barrier between the pharynx and the cervical esophagus. Three responses are implied by this definition: tone generation, phasic response activity, and sphincter opening. During swallowing, the pharyngoesophageal junction relaxes and opens, allowing foods and liquids to pass into the esophagus while acting as a barrier to retrograde flow. By doing so, it performs an important protective function, preventing aspiration of acidic gastric content into the respiratory tract on the one hand, and on the other—entry of air into the esophagus. The pharyngoesophageal junction also allows retrograde flow of material during belching and vomiting due to physiologic relaxation.

Not only anatomy but the treatment and prognosis of these tumors are intermediate between hypopharyngeal and esophageal tumors [45]. The diagnosis of this type of neoplasms varies depending on the circumstances of emergence and the neoplastic growth in this region. Pharyngeal–esophageal tumor symptoms are primarily characterized by selective dysphagia, for solid food at the beginning and total dysphagia afterwards, depending on the tumor evolution, but leading in the end to the alteration of the nutritional status [45]. This is often a sign of an advanced stage of this disease, but other symptoms are also present, such as weight loss, retrosternal pain, nausea, vomiting, dyspepsia, and anemia. Patients with pharyngoesophageal neoplasms often complain about paresthesia in the pharyngeal region, fetid halitosis, eructation, regurgitations, and foreign body sensation [45]. Metastases often develop in the liver, brain, lungs, and bones. According to HRQOL (health-related quality of life) questionnaires [46], the specific esophageal symptomatology persists or even deteriorates, leading to a poor quality of life in esophageal carcinoma survivors compared to the general population.

Squamous cell carcinomas in the hypopharynx (HP) and the cervical esophagus are two distinct diseases with different staging systems and treatment approaches. A pharyngoesophageal junction tumor involves both the hypopharynx and the cervical esophagus at the same time, but there have been few reports focused on pharyngoesophageal junction tumors. Although the hypopharynx and the cervical esophagus are anatomically adjacent, squamous cell carcinomas of the hypopharynx (HP) and cervical esophagus are distinct diseases with different staging systems and ways of treatment.

Cervical esophageal cancer is a very rare disease and is often locally advanced at the time of diagnosis, making local lesions difficult to control and survival rates low. The aggression of cervical esophageal carcinoma is high as it tends to grow in an abundant lymphatic drainage area and fails to produce early symptoms; it also easily and frequently extends towards the hypopharynx; these tumors are sometimes treated with schedules for locally advanced head and neck squamous cell carcinoma (LAHNSCC) which consist of 70 Gy in 35 fractions and 100 mg/m^2^ cisplatin on days 1, 22, and 43 of radiotherapy (RT) (The National Comprehensive Cancer Network guidelines for head and neck cancers); dCRT is related to life-threatening adverse events in 5–10% of patients; thus, further research is needed to define the optimal treatment schedule with adequate survival and acceptable toxicity [47].

Local failure of a neoplasm is a significant predictor of survival of cancer patients. Uno et al. [48] found that after definitive chemoradiotherapy (dCRT), none of the patients with initial local failure as determined by endoscopic examination survived more than 20 months compared with 2–5-year survival rates of 60% and 40%, respectively, in patients with initial local control. Local recurrences can be treated with saving surgery, which has a high morbidity rate, but it is the only option for relatively long-term survival. If not, palliative care options must be considered. Because of delayed diagnosis, poor performance of the majority of patients, and the high malignancy potential associated with particular anatomic characteristics, local and distant metastases occur frequently, with a 12–30% increased synchronous or metachronous risk, the survival rates for these kinds of patients being very poor. Cervical esophageal carcinoma is a very rare disease and often locally advanced at the time of diagnosis, resulting in limited locoregional disease control and poor survival. Finally, we need to recognize that optimal clinical support is needed to maintain dietary intake and exercise to optimize patient outcomes and quality of life. Cervical esophageal cancer is uncharted territory for many practitioners. Treatment of cancer in this region is often challenging due to its location in the cervical region, and most tumors are locally advanced cancers that have invaded the surrounding vital structures. To improve the survival outcomes and decrease the morbidity and mortality rates, future research should focus on the early detection of these cancers and improve treatment design by investigating innovative radiation schedules and identifying the optimal backbone for systemic therapy.

Clinical nutrition is required in the case of such patients as their nutritional status is assessed upon admission using body mass index protocols. Clinical nutrition must be maintained throughout the hospitalization while clinical parameters for each patient are evaluated (required caloric intake, biochemistry). If the digestive tract is functional, enteral nutrition via nasogastric tube, jejunal or gastric stoma with standardized nutritional supplements should be started as soon as possible [45]. The best medical approach to this disease is determined by a variety of factors, including general status, systemic and local implications, type of neoplasm, medical resources, and the acceptance of illness by the patient and their approval for medical attention [49,50]. One of the most important modern concerns in the management of this type of neoplasms is multimodal therapy that includes different medical and surgical specialties, also including, and very importantly, a psychologist.

Hebra was the first to recognize that skin pigmentation can indicate presence of visceral cancer in 1868 [51]. Since then, more than 50 dermatologic conditions have been identified as potential cancer markers [52]. Malignant diseases may involve the skin directly or indirectly. Direct involvement denotes the presence of tumor cells in the skin as a result of direct tumor extension or metastasis. In turn, indirect involvement is caused by a variety of factors, such as inflammatory, proliferative, or metabolic factors related to the neoplasm, such as polypeptides, hormones, cytokines, antibodies, or growth factors that act as mediators and interfere with cell communication and, thus, activity. There are no neoplastic cells in the skin in this case, so this involvement is classified as a dermatologic paraneoplastic syndrome [10,53].

At first glance, paraneoplastic skin manifestations may appear benign, and it is not always easy to establish a link between a dermatologic finding and an internal malignancy, let alone define the frequency of this association in the general population [52,53].

### 4.1. Bazex Paraneoplastic Acrokeratosis or Bazex Syndrome—Pharynx, Esophagus

In 1965, Bazex et al. [23] described the first patient with this condition as follows: “paraneoplastic syndrome with hyperkeratosis of the extremities”, because mainly it affects the nose, ears, hands, elbows, knees, and feet.

About 80% of cases are associated with upper aerodigestive tract tumors, such as of oral cavity, larynx, pharynx, trachea, esophagus, and lungs, and squamous cell carcinoma metastasis to cervical lymph nodes appears to be widespread in Bazex syndrome patients. In a retrospective study, the oropharynx and the larynx were involved in 48.6% of the cancers, followed by the lungs (17%) and the esophagus (10.6%) [10,15,54].

**Figure 1:**
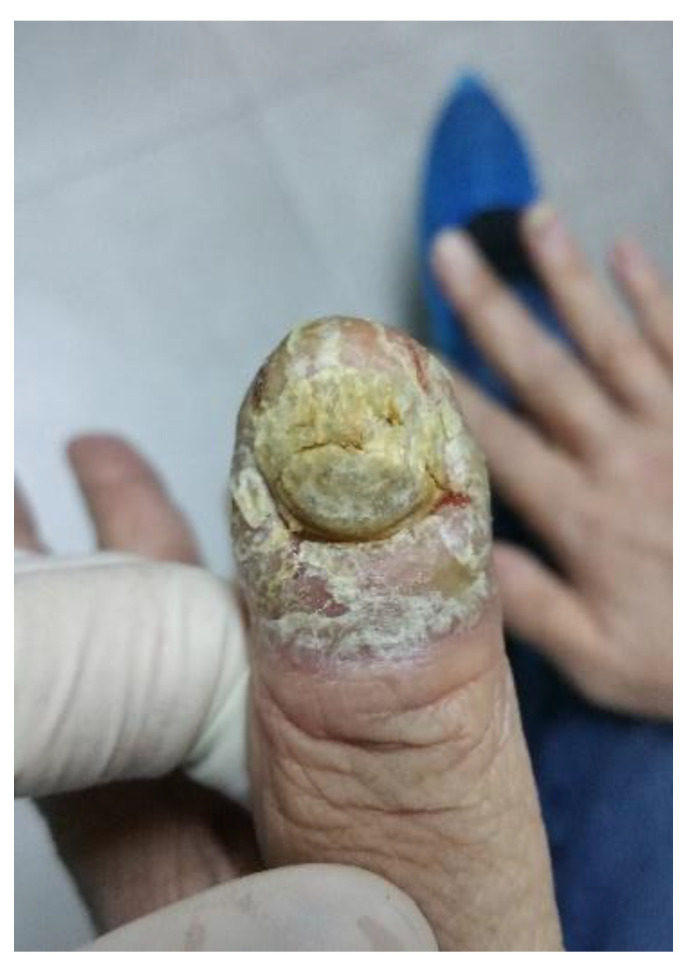
Bazex syndrome lesions.

**Figure 2:**
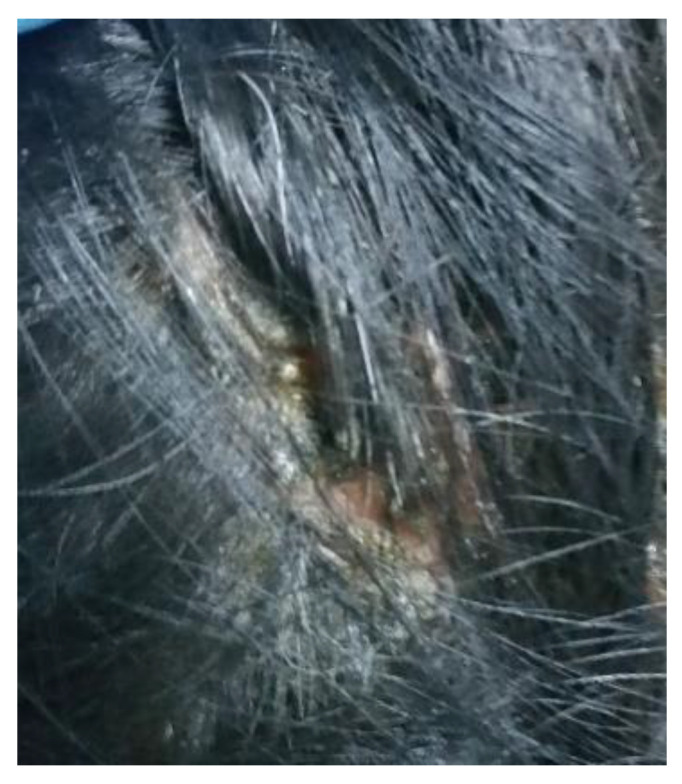
Bazex syndrome lesions.

**Figure 3:**
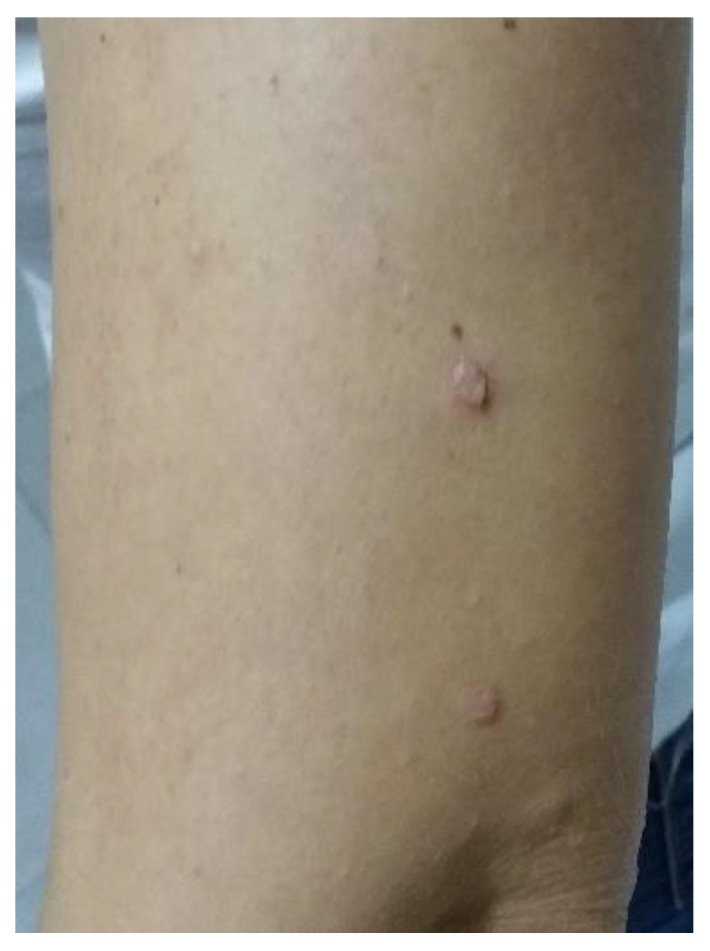
Bazex syndrome lesions.

It appears as symmetrical erythematous–violaceous scaly patches on the dorsum of the helix, nose, and distal ends of the extremities, with a psoriasiform aspect [54]. Desquamations occur in the dorsal and palmoplantar regions as the disease progresses, and nails are affected by subungual hyperkeratosis, dystrophy, and onycholysis (Figure 1,Figure 2,Figure 3). Other areas, such as the scalp, arms, knees, and legs, may be affected over time, with lesions spreading centripetally [24].

Bolognia et al. [14] reported the following findings in a retrospective study of the primary location of malignancies in 113 patients with Bazex syndrome: oropharynx and larynx, esophagus, lung, including one with an associated pyriform sinus carcinoma, and isolated cases of prostate, liver, stomach, vulva, bone marrow, and uterus.

### 4.2. Paraneoplastic Pemphigus—Hypopharynx, Esophagus

**Figure 4:**
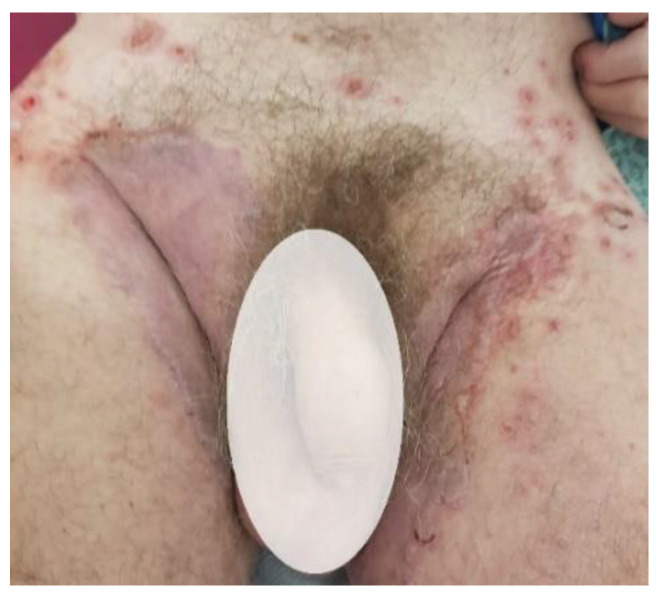
Pemphigus vegetans.

**Figure 5:**
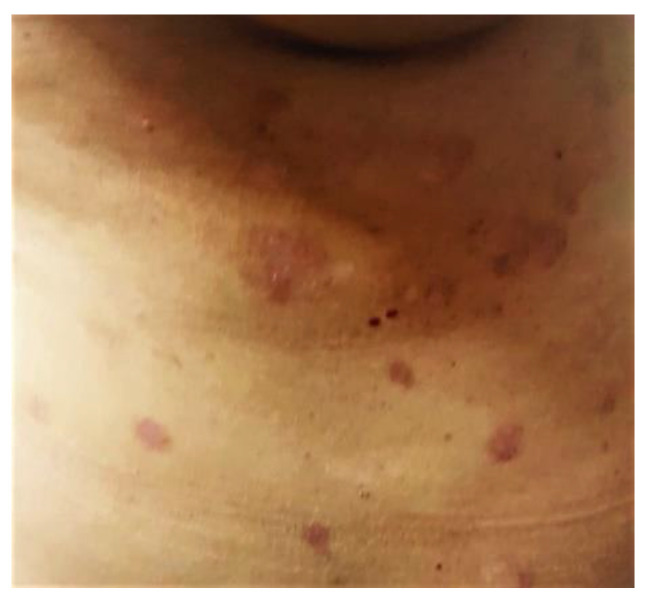
Pemphigus vulgaris.

**Figure 6:**
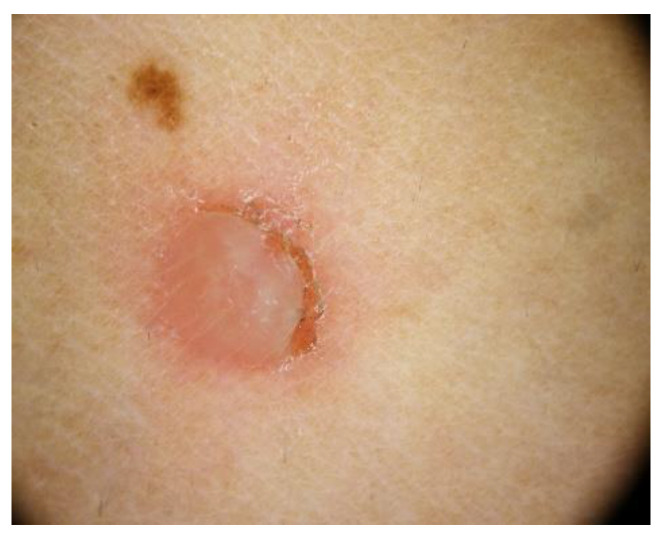
Pemphigus vulgaris—incipient phase.

Paraneoplastic pemphigus is considered a rare autoimmune disorder usually associated with confirmed or occult malignancy [55]. Patients typically present with extensive and painful mucosal and cutaneous involvement, usually having an overall poor prognosis [25,26]. Patients present with a variety of lesions with different morphologies, ranging from flaccid vesicles to extensive eruptions, some of which may be intensely itchy [25,27] (Figure 4,Figure 5,Figure 6). Paraneoplastic pemphigus always shows early mucosal involvement in the form of vesicles or bullae leading to painful mucocutaneous erosions and severe stomatitis, which may morphologically resemble pemphigus vulgaris [25,28].

Histologically, paraneoplastic pemphigus can resemble lichenoid eruptions (such as lichen planus, erythema multiforme drug eruptions), and other immunobullous diseases (pemphigus vulgaris, linear IgA bullous dermatosis, pemphigus foliaceous, IgA pemphigus, herpetiform pemphigus, drug-induced pemphigus). Correlation with clinical findings and immunofluorescence is invaluable in arriving at the correct diagnosis because etiopathogenetic differentiation can lead to a favorable prognosis [29].

Oral involvement with painful stomatitis is seen in almost all cases and can often be the first symptom, generally the least responsive to treatment. Oral lesions can be severe and diffuse, affecting the hypopharynx and the esophagus; they may also involve the conjunctival and anorectal mucosa. Skin manifestations range from erythema multiforme-like papules and plaques to pemphigus vulgaris-like vesicles and blisters and even lichen planus-like pruritic plaques [16]. In contrast to pemphigus vulgaris, acral and paronychial involvement is possible. Some patients develop respiratory complications such as bronchiolitis obliterans, which can lead to respiratory failure. Sepsis, hemorrhage, and respiratory failure are all associated with a high mortality rate in patients with paraneoplastic pemphigus [54].

### 4.3. Erythema Gyratum Repens—Esophagus

Erythema gyratum repens (EGR) is a paraneoplastic rash associated with a variety of malignancies and is considered one of the most prominent skin manifestations of solid tumors. EGR has a characteristic appearance consisting of undulating erythematous concentric bands that may be figural, circular, or annular [56,57].

Malignant neoplasms are found in 82% of patients with erythema gyratum repens [55,58,59]. Lung cancer is the most common (32%), followed by esophageal cancer (8%) and breast cancer (6%). Other malignancies such as colon, gastric, bladder, prostate, uterine, rectal, and pancreatic cancers, as well as multiple myeloma, have also been associated with erythema gyratum repens [30]. Approximately 80% of patients are diagnosed with erythema gyratum repens prior to the neoplasm, four to nine months prior to the diagnosis on average. Rarely, non-neoplastic diseases such as tuberculosis, pregnancy, calcinosis, esophageal dysmotility, sclerodactyly, Sjögren syndrome, and CREST syndrome may be associated with erythema gyratum repens [54,58].

### 4.4. Pityriasis Rotunda—Esophagus

The associated neoplasms include hepatocellular, gastric, and esophageal carcinoma, prostate cancer, chronic lymphocytic leukemia, and multiple myeloma [3]. Pityriasis commonly refers to flaking (or scaling) of the skin [55,59]. Although the conditions beginning with the name pityriasis have a different etiology, they do represent important dermatologic diseases, such as pityriasis versicolor or pityriasis folliculorum [59].

This disease is distinguished by multiple macules, which are circular, with hypo- or hyperpigmentation, usually found on the torso.

### 4.5. Palmoplantar Keratoderma—Esophagus

**Figure 7:**
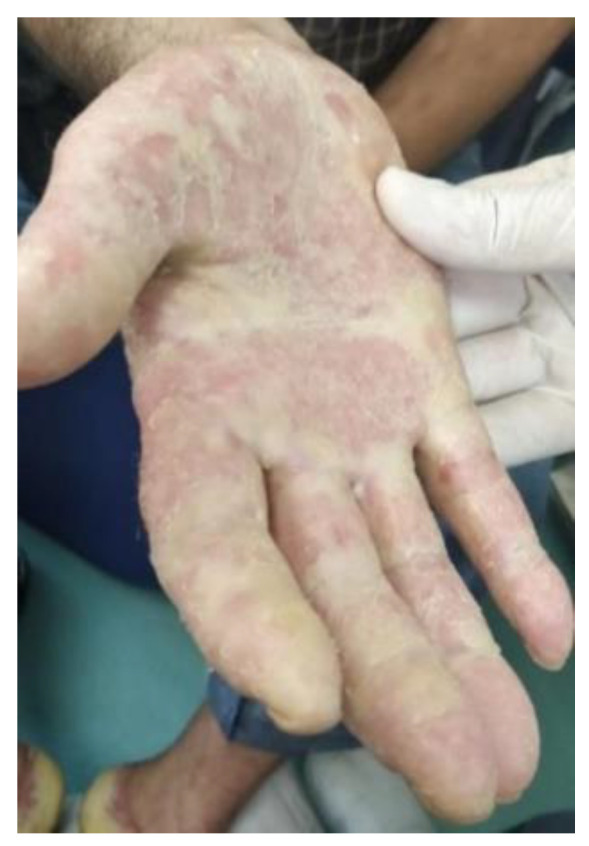
Palmar keratoderma.

**Figure 8:**
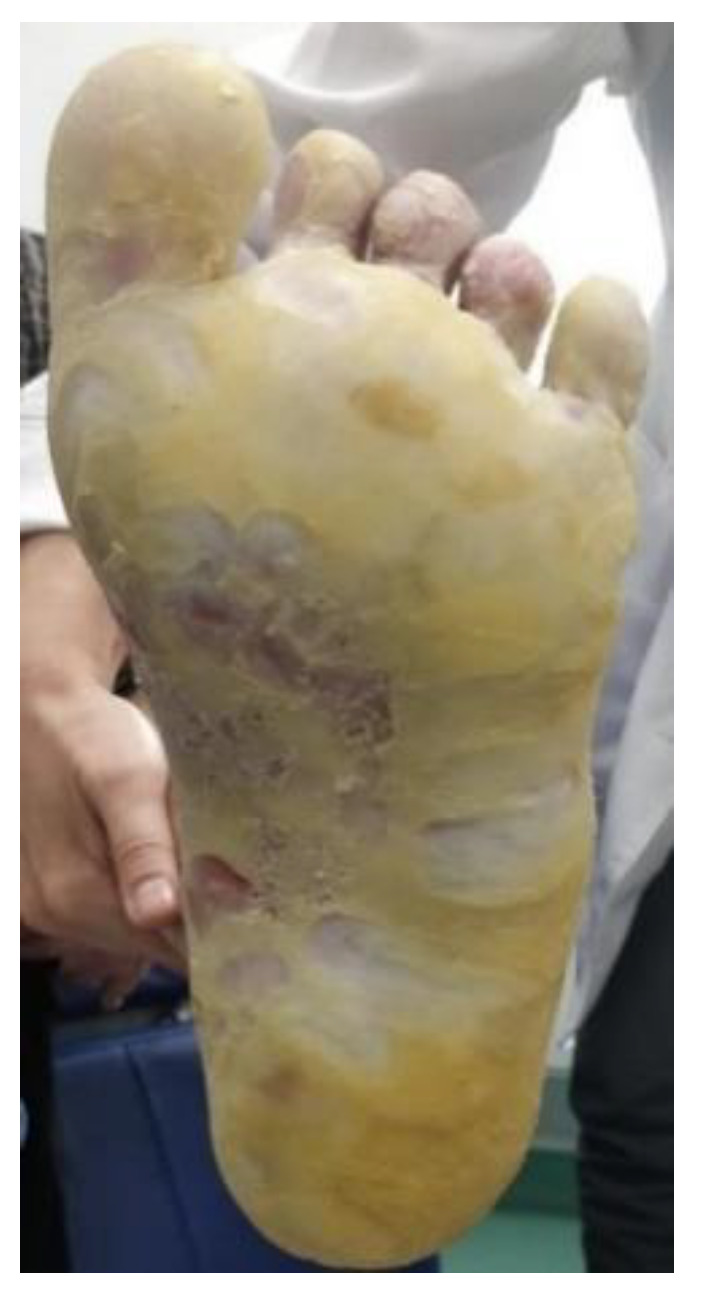
Plantar keratoderma.

It is a disease characterized by changes in keratinization that may be inherited or acquired (Figure 7,Figure 8). Several associations with malignancy have been described [55]. The prototype of the inherited disease is Howel–Evans syndrome, in which the risk of developing oral or esophageal carcinoma in increased 36-fold [55]. Skin lesions usually begin in childhood, although neoplastic involvement occurs at an average age of 61 years [10]. The pathogenesis of the syndrome has been linked to chromosome 17q24, a site where keratin is formed [12].

### 4.6. Paraneoplastic Dermatomyositis–Pharynx

Dermatomyositis is a rare idiopathic inflammatory myopathy that presents clinically with proximal muscle weakness and characteristic cutaneous manifestations [22].

Skin lesions can be classified as pathognomonic, characteristic, and compatible with dermatomyositis [31], but periorbital heliotropic rash and erythematous maculopapular lesions covering bony prominences are the more specific or pathognomonic manifestations of dermatomyositis [31,32]. The heliotropic rash appears as a red-to-purplish confluent macular erythema that affects the eyelids, upper cheeks, forehead, and temples symmetrically and is frequently associated with eyelid and periorbital tissue edemas [56].

In Southeast Asia, the incidence of nasopharyngeal carcinoma in men with or without dermatomyositis is increasing [18]. Another retrospective study described 12 patients with internal malignancy among 64 patients with polymyositis and 28 patients with dermatomyositis. Four of these 12 patients had malignancies of the gastrointestinal tract (two 74- and 75-year-old male patients had gastric carcinoma, another 51-year-old female had pharyngeal carcinoma, and one female had pancreatic carcinoma) [19]. Erosions of the oral cavity, pharynx, conjunctiva, gastrointestinal mucosa, and even anogenital area are examples of mucosal involvement [20,21].

### 4.7. Leser–Trelat Syndrome (LTS)—Esophagus

Ulysse Trelat (1884) and Edmund Leser (1901) [33] were the first surgeons to propose a link between internal malignancies and multiple seborrheic keratoses. There is no evidence of dermatitis or erythroderma prior to the appearance of seborrheic keratoses on the skin, and pruritus is a leading symptom in approximately half of the cases [34].

**Figure 9:**
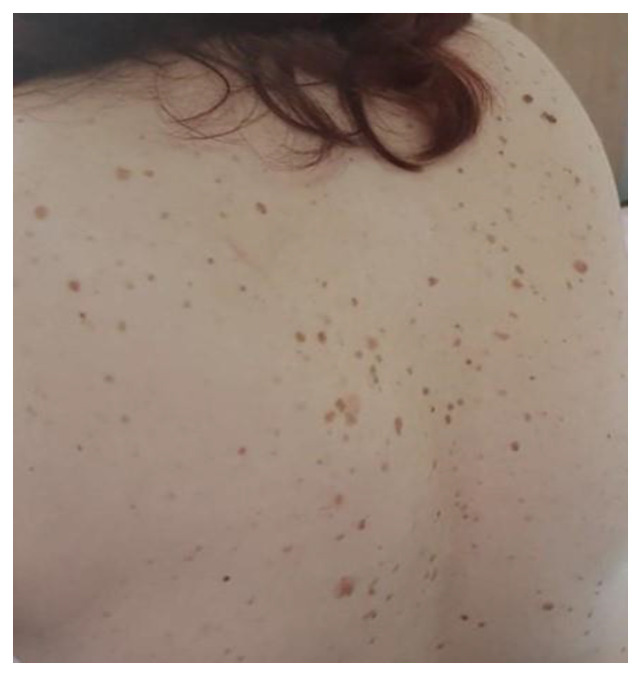
Leser–Trelat syndrome.

This syndrome is a relatively rare clinical condition found to be associated with internal malignancies and characterized by the sudden and eruptive appearance of multiple seborrheic keratoses in association with underlying malignant disease [56] (Figure 9).

Most patients with LTS have adenocarcinomas, most commonly of the stomach [35,55], colon, or rectum [36,37,38,39,40,41,42], or, less commonly, carcinomas of the esophagus [22], duodenum [43], pancreas [44], gallbladder [4], or hepatocellular carcinoma [60,61,62].

Finally, 80% of the patients with paraneoplastic dermatoses found at our clinic, filtering for the pharyngoesophageal malignancies, were the ones with esophageal neoplasms, out of which seven were female and two were male, making esophageal cancer more common within paraneoplastic dermatoses within pharyngoesophageal malignancies.

## 5. Conclusions

Numerous systemic diseases can be diagnosed through the skin, including changes suggestive of internal malignancies. Cutaneous paraneoplastic syndromes are important clinical markers that may precede, co-occur with, or follow the diagnosis of a specific neoplasm. Plenty of dermatoses have been correlated with underlying neoplastic processes, many of which correlate with specific neoplasms, thus providing an important diagnostic aid. Skin manifestations suggestive of a malignancy are extremely useful in making a definitive early diagnosis. The knowledge of paraneoplastic syndromes is essential for first-line clinicians to improve the prognosis and the quality of life of patients. The data presented may contribute to the development of continuing postgraduate education programs for physicians of various specialties.

## Figures and Tables

**Table 1:** Skin manifestations in pharyngoesophageal malignancies.

Author	Dermatological Manifestation	Neoplastic Topography
Thiers et al., 2009 [10] Lee, 2009 [15] Dourmishev and Draganov, 2009 [11] Bolognia et al., 1991 [14] Bazex et al., 1965 [23] Ljubenovic et al., 2009 [24]	Bazex paraneoplastic acrokeratosis/ Bazex syndrome	Upper aerodigestive tract/ pharynx/esophagus
Pipkin et al., 2008 [3] Ehst et al., 2010 [12] Edgin et al., 2008 [16] Boyce and Harper, 2002 [17] Dourmishev and Draganov, 2009 [11] Kartan et al., 2017 [25] Helm et al., 1993 [26] Kimyai-Asadi et al., 2001 [27] Choi et al., 2012 [28]	Paraneoplastic pemphigus	Hypopharynx/esophagus
Ramos-E-Silva et al., 2011 [2] Pipkin et al., 2008 [3] Dourmishev and Draganov, 2009 [11] De La Torre et al., 2011 [29] Serrao et al., 2008 [30]	Erythema gyratum repens	Esophagus
Pipkin et al., 2008 [3] Dourmishev and Draganov, 2009 [11]	Pityriasis rotunda	Esophagus
Thiers et al., 2009 [10] Ehst et al., 2010 [12] McLean, 1987 [13] Dourmishev and Draganov, 2009 [11]	Palmoplantar keratoderma	Esophagus
Leow and Goh, 1997 [18] Wakata et al., 2002 [19] Joly et al., 2000 [20] Anhalt, 2004 [21] Dourmishev and Draganov, 2009 [11]	Paraneoplastic dermatomyositis	Pharynx
Tutakne et al., 1983 [22] Leser et al., 1901 [31] Swartz et al., 1991 [32] Yeh et al., 2000 [33] Kameya et al., 1988 [34] Cohn et al., 1993 [35] Hodak et al., 1987 [36] Liddell et al., 1975 [37] Heng et al., 1988 [38] Brauer et al., 1992 [39] Ginarte et al., 2001 [40] Klimopoulus et al., 2001 [41] Ohashio et al., 1997 [42] Kocygit et al., 2007 [43] Tajima et al., 1991 [44]	Leser–Trelat sign	Esophagus

## Data Availability

Not applicable.
